# Theory and data for simulating fine-scale human movement in an urban environment

**DOI:** 10.1098/rsif.2014.0642

**Published:** 2014-10-06

**Authors:** T. Alex Perkins, Andres J. Garcia, Valerie A. Paz-Soldán, Steven T. Stoddard, Robert C. Reiner, Gonzalo Vazquez-Prokopec, Donal Bisanzio, Amy C. Morrison, Eric S. Halsey, Tadeusz J. Kochel, David L. Smith, Uriel Kitron, Thomas W. Scott, Andrew J. Tatem

**Affiliations:** 1 Fogarty International Center, National Institutes of Health, Bethesda, MD, USA; 2 Department of Entomology and Nematology, University of California, Davis, CA, USA; 3 Emerging Pathogens Institute, University of Florida, Gainesville, FL, USA; 4 Department of Geography, University of Florida, Gainesville, FL, USA; 5 Department of Global Health Systems and Development, Tulane University School of Public Health and Tropical Medicine, New Orleans, LA, USA; 6 Department of Environmental Sciences, Emory University, Atlanta, GA, USA; 7 United States Naval Medical Research Unit No. 6, Lima, Peru; 8 Department of Epidemiology, Johns Hopkins Bloomberg School of Public Health, Baltimore, MD, USA; 9 Malaria Research Institute, Johns Hopkins Bloomberg School of Public Health, Baltimore, MD, USA; 10 Department of Geography and Environment, University of Southampton, Southampton, UK; 11 Flowminder Foundation, Stockholm, Sweden

**Keywords:** activity space, agent-based model, co-location and contact networks, human mobility, simulation, synthetic population

## Abstract

Individual-based models of infectious disease transmission depend on accurate quantification of fine-scale patterns of human movement. Existing models of movement either pertain to overly coarse scales, simulate some aspects of movement but not others, or were designed specifically for populations in developed countries. Here, we propose a generalizable framework for simulating the locations that an individual visits, time allocation across those locations, and population-level variation therein. As a case study, we fit alternative models for each of five aspects of movement (number, distance from home and types of locations visited; frequency and duration of visits) to interview data from 157 residents of the city of Iquitos, Peru. Comparison of alternative models showed that location type and distance from home were significant determinants of the locations that individuals visited and how much time they spent there. We also found that for most locations, residents of two neighbourhoods displayed indistinguishable preferences for visiting locations at various distances, despite differing distributions of locations around those neighbourhoods. Finally, simulated patterns of time allocation matched the interview data in a number of ways, suggesting that our framework constitutes a sound basis for simulating fine-scale movement and for investigating factors that influence it.

## 1. Introduction

The importance of mathematical modelling of human movement is far ranging, as movement patterns underlie key issues in public health, economics and urban planning [1,2]. Different questions in each of these arenas, however, necessitate the selection—and sometimes the development—of models with different levels of spatial and temporal resolution.

In infectious disease epidemiology, describing average flows of movement between cities is informative of the spread and persistence of pathogens over broad geographical areas [3–5]. At more local scales, such as within a city, movements by smaller groups must be acknowledged to capture structure relevant to the dynamics of transmission [6–9] and the uniqueness of exposures of those different groups [10]. Different types of movement over different time scales must also be considered. Migratory and seasonal movements are likely important for circulation over broad regions and time scales, whereas commuting patterns and other routine movements give rise to the structure of contacts on which epidemics spread [1,11].

For modelling movement at fine spatial scales, such as within cities, many models (e.g. [12–14]) focus on scales no finer than several hundred square metres, or the area covered by a mobile phone tower, because that represents the finest scale at which an individual's presence can be deduced with mobile phone call records. In certain locations, however, data informative of an individual's whereabouts at finer scales could be used to motivate and parametrize models at the scale of buildings or lots [15,16]. An existing conceptual framework for modelling movement at this scale is that of an individual's activity space, which is defined as ‘the subset of all urban locations with which the individual has direct contact as the result of day-to-day activities’ [17].

Modelling the composition of activity spaces and movement within them is essential for any simulation of synthetic human populations in an urban environment. Such simulations are of high utility for a number of applications in infectious disease epidemiology, including planning for the containment of influenza or smallpox outbreaks [18–21] and evaluating the efficacy of a putative dengue vaccine [22,23]. Although these and other applications are of relevance to cities in the developing world, the development of algorithms for simulating the composition and dynamics of activity spaces has focused primarily on cities in North America and Europe. Furthermore, simulation models of human activity spaces typically focus on details that, while important for applications in transportation [24], may be unnecessarily complex for application to the epidemiology of many infectious diseases [15]. Still other models are capable of simulating movement and time allocation within an activity space (e.g. [25,26]), but provide no basis for simulating which locations comprise the activity space. A generalizable framework that can be parametrized with readily attainable data and that can be used to simulate activity space composition and time allocation in a variety of geographical contexts is therefore needed.

**Figure 1.**
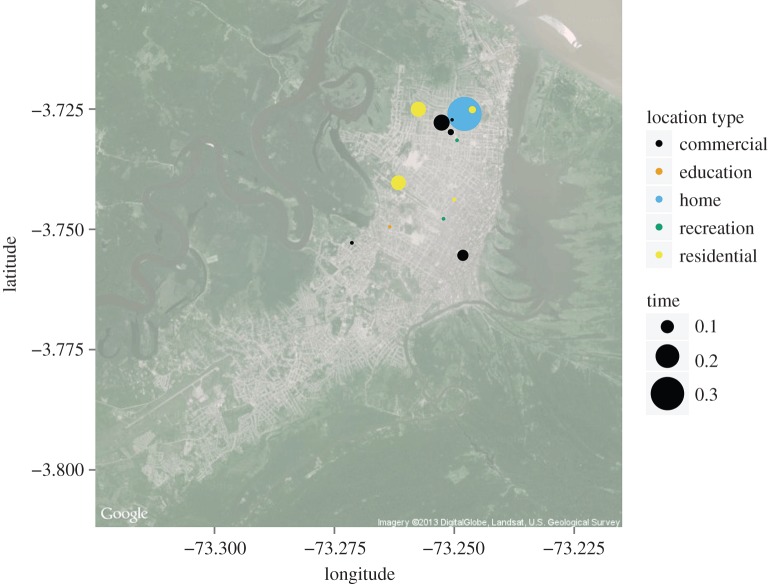
Example of an individual's activity space and the proportion of the individual's time spent at each location, simulated with the fitted model. (Online version in colour.)

To address this need, we developed a modelling framework that integrates five distinct aspects of movement—i.e. number of locations in the activity space, location type and distance from home of locations in the activity space, and frequency and duration of visits to those locations—to generate a cohesive description of time allocation within an individual's activity space. This framework allows for simulation of locations comprising the activity space and parameters that govern a stochastic process of movement between pairs of locations within the activity space. Together, this results in a description of how the individual allocates time across the activity space. To demonstrate the utility of this framework, we fit a model of activity space composition and time allocation to data from retrospective interviews of 157 residents of the city of Iquitos in northeastern Peru. Using these data, we selected among candidate models with varying levels of detail about location type and distance from home, and we used simulations of the best-supported model (e.g. figure 1) to assess the model's ability to reproduce empirical patterns of time allocation in the study population.

## 2. Material and methods

### 2.1. Modelling framework

#### 2.1.1. Activity space composition

**Figure 2.**
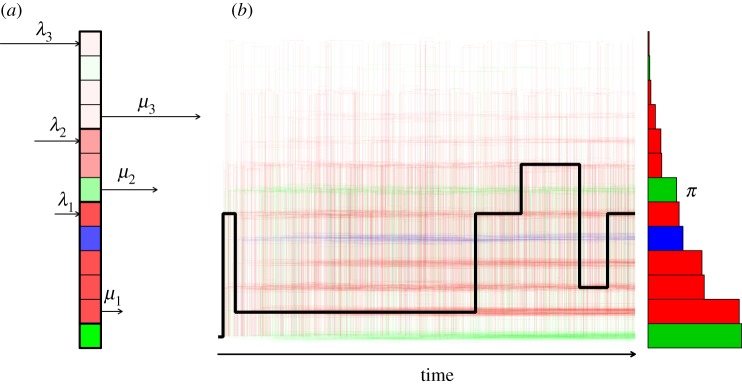
Model schematic. (*a*) Snapshot of an individual's activity space at a given point in time, with the individual's home on the bottom, a relatively permanent class above it, an intermediate class above that, and a relatively transient class at the top, with classes separated by thick lines. The composition of each class is governed by an M/M/1 queue with ‘birth’ rate *λ*_*i*_ and ‘death’ rate *μ*_*i*_. Different colours indicate different location *types* (e.g. houses, shops, parks), which are distinct from the permanent, intermediate and transient location *classes*. (*b*) An individual's whereabouts over time, with a single example in black and 500 replicates in faint colours. On average, the overall proportion of time spent at each location tends to the distribution *π*. (Online version in colour.)

The first mathematical characteristic of an individual's activity space that we define is that it is comprised of locations belonging to *m* different classes, each of which is distinguished by how long locations of that class tend to remain in the individual's activity space. For example, many people likely have some locations that they visit routinely (e.g. relatives' houses) as well as some that they do not visit as a matter of routine (e.g. repair shops, airports). We posit that routine locations remain in one's activity space for long periods of time, whereas irregular locations come and go from the individual's activity space over time. Each such class *i* is defined by a constant rate *λ*_*i*_ at which new locations are added to it and a constant rate *μ*_*i*_ at which a location of that class is removed from the activity space (figure 2*a*). If we assume that locations in each class are removed from the activity space in the same order in which they were added to it, then each class within an individual's activity space can be modelled as a queue (of M/M/1 type [27]). This convenience means that we can directly calculate some key characteristics of the dynamics of each class, including the stationary distribution of the number of locations of each class in the activity space (geometric with parameter *ρ*_*i*_ = 1−*λ*_*i*_/*μ*_*i*_) [27, pp. 548–552]. The stationary distribution of the number of locations across all classes in the activity space is thus a sum of *m* geometric random variables. In the event that *m* is finite, the stationary distribution of activity space size follows a negative binomial distribution with parameters *m* and *ρ* if all *ρ*_*i*_ = *ρ*. If there is a very large number of classes (i.e. as *m* → ∞), the stationary distribution of activity space size is a Poisson distribution with parameter *mρ*. Finally, in the trivial case with only a single class, the stationary distribution of activity space size is geometrically distributed with parameter *ρ*.

#### 2.1.2. Time allocation within the activity space

Because there is potential for locations to be added to or removed from the activity space at any time, we complement the continuous-time process of activity space turnover with a continuous-time process of movement by an individual within her or his activity space. A simple and general way to model this movement process is with a continuous-time, finite-state Markov process (figure 2*b*), similar to how geographers have modelled trip behaviour [28,29]. Under such a formulation, the set of *n* locations in an individual's activity space comprise the states among which an individual moves about according to the *n* × *n* rate matrix *Q* [27, p. 396]. Each off-diagonal element *q_i_*_,*j*_ of *Q* represents the instantaneous probability that an individual moves from location *i* to location *j*, and the elements of this matrix are stipulated to satisfy the condition that *q_i_*_,*i*_ =−∑*_j_*_≠i_ *q_i_*_,*j*_. Characteristics of a movement process obeying these dynamics include that the durations of visits to each location *i* are exponentially distributed with mean −1/*q_i_*_,*i*_ and that the stationary distribution *π*, which is a vector containing the long-term average proportion of one's time spent at each location, satisfies *πQ* = 0 [27, pp. 395 and 398].

### 2.2. Model refinements

Applying this framework to specific populations requires specifying which locations are included in an individual's activity space and how an individual spends time at and moves among those locations. Here, we investigated two primary characteristics of locations—location type and distance from home—that affect whether individuals visit those locations and if so how frequently and for how long they visit (table 1).

**Table 1.** Summary of model components, model subcomponents and how variation in each is represented probabilistically in the best-supported model.

model component	model subcomponent	probability distribution
locations visited	number of locations	negative binomial (*r*, *ρ*)
	locations of a given type	multinomial (*p_*τ*_*)
	locations of a given distance	∝ pdf(*δ*; *τ*) · exp(−*μ*_*τ*_*δ*^*η*_*τ*_^)
time allocation	frequency of visits	lognormal (*μ*(*δ*; *a_d_*_,*τ*_, *b_d_*_,*τ*_, *c_d_*_,*τ*_), *σ*_*d*__,*τ*_)
	duration of visits	lognormal (*μ*(*δ*; *a_f_*_,*τ*_, *b_f_*_,*τ*_, *c_f_*_,*τ*_), *σ*_*f,τ*_)

#### 2.2.1. Location types

Given some way of classifying locations according to *T* types, such as residential or commercial, we propose that whenever a new location is added to one's activity space it has a probability *p*_τ_ of being a location of type *τ*, where ∑*p*_τ_ = 1. Consequently, the number of locations of each type in an activity space comprised of *n* locations is a multinomial random variable with parameters *n* and 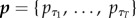. These location types are not necessarily the same as the location classes. Rather, we envision location types as readily distinguishable based on observable characteristics (e.g. houses, schools) and location classes as defined solely by the extent of their transience in one's activity space (e.g. a workplace and a market could belong to the same class).

#### 2.2.2. Distance from home

We also consider the possibility that individuals tend to display a preference for locations that are closer to their homes. To investigate this possibility, we must first consider how far away locations of a certain type are from one's home. If an individual chooses locations randomly with respect to distance from home, then distances at which more locations of a given type are present would be more likely to be chosen. Thus, we must first account for the distribution *p*(*δ*; *τ*) of locations of type *τ* at various distances *δ* and then determine whether the realized distribution of locations visited is weighted in favour of those closer to home. We do so with a weighting function of the form2.1
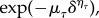
which is similar to an exponential distribution with rate *μ* but with an additional parameter *η* to allow for more flexibility to weight locations very close to home more strongly. Once the distance from home of a new location is determined, we select a specific location randomly from all locations of a given type *τ* at that distance. We furthermore consider the possibility that different location types have different weighting functions, as determined by the parameters *μ*_*τ*_ and *η*_*τ*_.

#### 2.2.3. Time allocation

To determine how individuals allocate time across locations in their activity spaces, we assign each individual a frequency of visits, *f_i_*, and a mean duration of each visit, *d_i_*, to each location *i* in the activity space. The collection of all *f_i_* and *d_i_* for 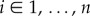 is then used to populate the matrix *Q*. Because the diagonal entries of *Q* are directly related to the mean duration of visits to each location, these entries are defined simply as *q_i_*_,*i*_ =−1/*d_i_*. To populate the off-diagonal entries of *Q*, which describe movements between locations, we herein make the simplifying assumption that consecutive movements are uncorrelated and proportional to the frequency at which an individual visits each location, such that2.2
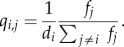
To select *f_i_* and *d_i_* for different individual–location pairs, we model these parameters as bivariate normal random variables on a natural log scale with means *μ*_*f,τ*_ and *μ*_*d,τ*_, standard deviations *σ*_*f,τ*_ and *σ*_*d,τ*_, and correlation *ρ*_*τ*_. Moreover, we consider the possibility that the means and standard deviations differ for different location types and that the means depend on distance from home according to the function2.3
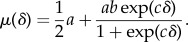
This function is defined such that the mean frequency or duration of visits on a natural log scale to a location of a given type *τ* approaches (1*/*2)*a*(1 + *b*) near home and (1*/*2)*a* as *cδ* → −*∞*. Finally, we assume that the frequency and mean duration of visits to one's own home and to locations outside the city are also jointly distributed lognormal random variables but that they are characterized by their own parameters, which do not depend on *δ*.

### 2.3. Data

The data used to fit the model were collected in the Amazonian city of Iquitos, Peru. Iquitos is an isolated city of approximately 377 000 inhabitants surrounded on three sides by rivers. Only one regional road leaves the city, meaning that most local travel outside the city takes place by boat. Besides walking short distances, movement within the city typically occurs by motorcycle, motorcar or bus. Neighbourhoods within the city vary in the availability of services and quality of construction. Most contain key services (schools, health centres, markets), but specialized commerce and other services are concentrated in specific areas of the city (e.g. two main hospitals, a shopping district in the downtown area). Participants in this study lived in either of two neighbourhoods (electronic supplementary material, figure S1), Maynas and Tupac Amaru, which exhibit modest differences in housing construction and population density [30]. These neighbourhoods were initially chosen for other studies because they are mostly self-contained (having schools and health centers) and are distant enough to limit inter-neighbourhood movement [9,30]. Historically, dengue virus transmission and mosquito densities have been higher in Maynas [31].

#### 2.3.1. Geographical information system

Construction of a geographical information system (GIS) for the city of Iquitos began in 1998 [32] and was ongoing at the time of this study [30,31,33]. We used coverages that encompassed nearly 50 000 lots using the coordinate system Universal Transverse Mercator WGS-84 Zone 18S. In areas with ongoing epidemiological and entomological research projects, field personnel assigned location types to each lot. It should be noted though that many sites were mixed use; e.g. residential and commercial in many cases. During the retrospective interview study, unmapped locations were physically located to obtain Geographical Positioning System coordinates and to update the GIS.

#### 2.3.2. Retrospective interviews

Retrospective interviews were conducted using a semi-structured interview (SSI) [34,35]. The SSI was developed to address the issues of recall, reliability, reproducibility, compliance, behavioural change and privacy that are typically associated with classic movement survey methods, such as interviews, diaries or direct observation. This tool was designed for use with people who became infected with dengue or who were in shared spaces with people who had become infected with dengue (potential future dengue cases), to find out where they had been in the past two weeks (to identify potential exposure sites). Hence, we were limited to methods that could be applied retrospectively. Based on focus groups conducted to develop the SSI, we found that: (i) people could identify routine locations they visit, but triggers were needed for certain types of locations, (ii) the best aid for recalling locations visited was to begin the SSI by thinking about a ‘typical day’ and to gradually add memory triggers over the course of the interview, and (iii) there were clear ‘common activity spaces’ identified for all. During the development and validation of the SSI, we found that high-resolution satellite imagery of participants' neighbourhoods, combined with street labels, was not very useful in triggering recall of locations, but aided participants in describing and locating where they had been. If participants were unable to find a location on the map, they would either call someone at the location to ask for the address, or give our research team the best description they could. Our research team would then find the location and record its position. The SSI followed with a section on common activity spaces already identified by others (e.g. schools, markets, health facilities). The SSI concluded with a section that listed categories of locations that tended to require triggers to be recalled or else would otherwise likely go unreported. The result of each interview was a list of the locations that an individual visited in the two-week period preceding the interview, as well as estimates of the frequency and duration of visits to each location.

A total of 120 participants provided information about time spent at home, 138 about time spent elsewhere and 101 of 157 provided information about both. These interviews were conducted with residents of two neighbourhoods (electronic supplementary material, figure S3), from which study participants were obtained by convenience sampling and are thus not completely representative of the local population in terms of age, sex, occupation and possibly other factors; they are nonetheless diverse (electronic supplementary material, figures S2–S4). Interviews were mostly conducted during the dengue transmission season (electronic supplementary material, figure S5), which spans several months, as well as variation in seasons that could impact movement behaviour (e.g. times when children are in or out of school).

### 2.4. Analysis

#### 2.4.1. Model fitting and selection

The first aim of our analysis was to fit and select among candidate models for each of the five aspects of movement. For example, candidate models that we considered for the distribution of the number of locations visited included geometric, Poisson and negative binomial distributions. Candidate models for all aspects of movement are listed in table 2. For each aspect of movement, each candidate model was fitted separately to the interview data by numerically estimating maximum-likelihood parameter values. To compare a pair of models with nested parameters, we performed a likelihood ratio test and selected the more complex model if *p* < 0.05. For situations with more than two candidate models, we assessed the relative support for each by computing each model's Akaike information criterion corrected for finite sample size (AIC*_c_*), which balances goodness of fit and model complexity, and then Akaike weights, which are a measure of relative support [36].

**Table 2.** Summary of candidate models for each model subcomponent.

model subcomponent	candidate models
number of locations	geometric, Poisson, negative binomial
locations of a given type	all possible location type groupings in which types within a group have identical probabilities of being found in an activity space
locations of a given distance	all possible location type groupings in which types within a group have identical effects of distance from home, which depend on either one or two parameters per location type grouping
frequency of visits	all possible location type groupings in which types within a group have identical effects of distance from home or no effect of distance from home. Zero or non-zero correlation with duration of visits
duration of visits	all possible location type groupings in which types within a group have identical effects of distance from home or no effect of distance from home. Zero or non-zero correlation with frequency of visits
time at home	zero or non-zero correlation between frequency and duration of times at home

To explore the set of possible models with different levels of detail about location type, we employed a form of backward elimination. To do so, we first fitted the candidate model with the finest breakdown of location types that we considered, such that each location type had its own set of parameters. We then chose the pair of location types with the most similar parameter values and fitted a new model in which the parameters of those two location types were constrained to be equal. Repeating this procedure of agglomerating location types based on parameter similarity, we obtained a set of candidate models with a range of location-type categorizations, from the case in which each location type had its own parameter values to the case in which all location types had the same parameter values. Additional details of the model selection procedure specific to different aspects of movement are elaborated on in the electronic supplementary material, table S2.

For all aspects of movement, we also fitted models separately to interviews of residents of two distinct neighbourhoods in Iquitos to assess the robustness of our fitted model to possible neighbourhood-specific differences (which have been found elsewhere [37]). In doing so, we applied the same model selection procedures as described above, and we assessed support for either the aggregated model (denoted *M*_*M* +*T*_) or the disaggregated model (*M_M_* + *M_T_*) by ΔAIC*_c_* > 10 (as recommended for a pair of non-nested models [36, p. 123]).

#### 2.4.2. Comparison of model outputs against data

To assess the realism of patterns of time allocation simulated with our model, we compared simulation results against patterns of time allocation derived directly from the retrospective interviews. Although data from these interviews were also used to parametrize the model, the model's ability to reproduce patterns of individual time allocation does not necessarily follow, because the features of the interview data with which the model was parametrized are distinct from those against which model outputs were compared. This comparison therefore allowed us to assess the model's ability to translate basic aspects of movement into descriptions of time allocation across locations, which is the objective of the model and a goal that is common to many applications in epidemiology and other fields.

To that end, we first calculated the empirical pattern of time allocation within each individual's activity space. For the same number of individuals as participated in retrospective interviews of time spent at home and elsewhere (*n* = 101), we simulated patterns of time allocation 10^3^ times (e.g. figure 1). Given these empirical and simulated patterns of time allocation, we then examined (i) the proportion of individuals that allocated a certain proportion of their time at a single location of each location type as well as at their home and (ii) how time allocation was distributed over distance from home.

Our quantitative approach to the comparison of simulated and empirical patterns of time allocation was based on that of statistical hypothesis testing. For a given statistic (e.g. proportion of time allocated at a distance of 1 km from home), the set of simulated values of that statistic comprised the null distribution against which the empirical value was compared. Formally, and assuming a two-tailed test, if the empirical value of a given statistic fell between the 2.5th and 97.5th percentiles of this null distribution, then our interpretation was that we could not reject the possibility that the empirical value was generated by the process codified by our model. Informally, given that any single statistic that we considered fell along a continuum of related statistics (e.g. distances from home of 100 m, 200 m, etc.), we found it instructive to visually compare how well the entire set of empirical statistics tracked the set of simulated statistics.

## 3. Results

### 3.1. Model fitting and selection

#### 3.1.1. Activity space composition

**Figure 3.**
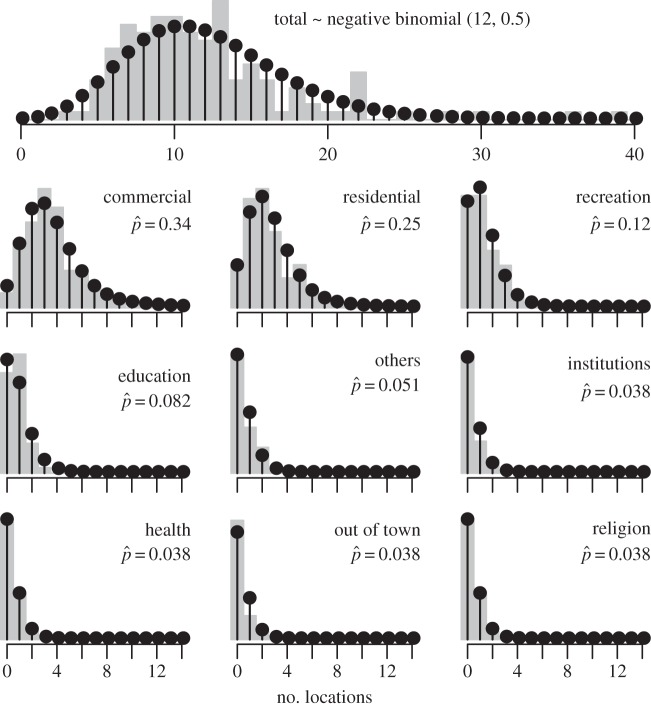
Numbers of locations in individuals' activity spaces, in total (top) and by location type (other panels). Grey bars show empirical data from retrospective interviews (*n* = 138). Black stems show a fitted negative binomial distribution (top) and a fitted multinomial distribution conditioned on the empirical distribution of total numbers of locations and marginalized for each location type (other panels).

Among the three distributions that we considered, we found clear support for the negative binomial (Akaike weight ≈ 1, table S3, figure 3). This result is consistent with the hypothesis that activity spaces comprised *m* = 12 classes each with identical parameter *ρ* = 0.4997, or, alternatively, that activity space size was Poisson distributed with variation among individuals' parameters that was gamma distributed with hyperparameters equal to *r* and *ρ*/(1 − *ρ*).

We found support for uneven representation of location types within individuals' activity spaces, with the best-supported model being those in which there were six distinct location-type groups, and with models with between five and nine such groups having nearly all Akaike weight (table S4). The best-supported model assigned 34% of locations as commercial, 25% as residential, 12% as recreational and others at less than 10% each (figure 3). All models with some fitted vector ***p*** fit the data better than a model in which ***p*** equalled the proportions of locations of each type within the city (table S4), which represented the hypothesis that locations were chosen irrespective of type.

**Figure 4.**
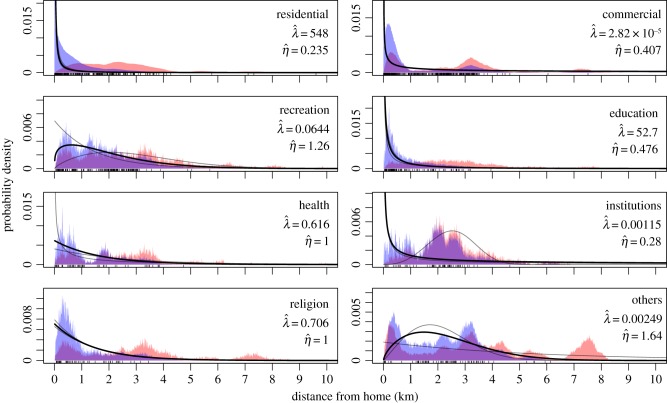
Relationship between distance from home and the probability that a location of a given type is chosen for one's activity space. The probability density of distances from home at which activity space locations of a given type are located (blue area) is obtained by weighting the probability density of distances from home at which all locations of a given type are located (red area) by a function for how distance from home affects the probability of being chosen for one's activity space (curves). The thick black curves (whose parameter values are listed in each panel) were fitted to all interview data without consideration of the neighbourhood in which interviewees live, whereas the thin grey curves were fitted separately to interviews from residents of the Maynas and Tupac neighbourhoods. Rug plots along the bottom of each panel indicate the distances from home at which locations in the activity spaces of study participants were located. (Online version in colour.)

For each location type that we considered, there was a distinct effect of distance from home on its inclusion in one's activity space, as the model with separate parameters for each location type had an AIC*_c_* value 49 lower than the next best model (table S5). For locations in the recreation or others categories, individuals were more likely to visit locations of an intermediate distance from home (approx. 0.5−2 km) than they were locations nearby or far away (figure 4). For all other location types, individuals were less likely to visit locations farther from home, with this effect being particularly strong for locations in the residential, commercial, education and institutions categories (figure 4). These different relationships for different location types are likely attributable to aspatial considerations, given that we accounted for the spatial distribution of each location type relative to study participants' homes.

#### 3.1.2. Time allocation

**Figure 5.**
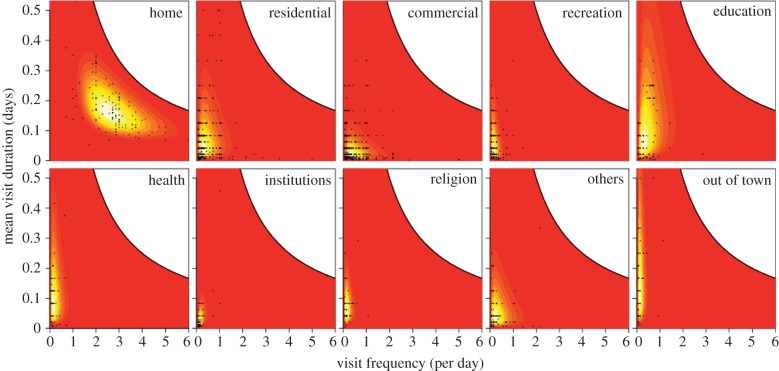
Joint distributions of the frequency and mean duration of visits to one's own home, to locations of various types within Iquitos, and to locations outside the city. Data from retrospective interviews (black dots) are superimposed on bivariate lognormal probability density surfaces (relative density: red, low; orange, intermediate; yellow, high) with maximum-likelihood parameter estimates. Note that (i) surfaces for residential and commercial location types reflect maximum-likelihood parameter estimates at a distance of 100 m from home and that other distances have somewhat different surfaces and (ii) it is not possible for data to occupy the white region above the black curve, which represents the combinations of frequencies and mean durations that result in a person spending all of their time at a single location. (Online version in colour.)

The frequency and mean duration of visits to home were best described by a bivariate lognormal distribution with an average frequency of 2.79 times per day, an average mean duration of 4.2 h per visit (between the hours of 5.00 and 22.00), and a significant negative correlation between those quantities (*ρ* = −0.59; likelihood ratio test: 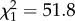, *p* = 6.2*e* − 13) (figure 5).

For locations other than home, one attribute that we found to affect both the frequency and mean duration of visits was location type. Comparing models of the frequency and mean duration of visits with different numbers of location-type groups, we found strong support for the model with the most detailed representation of location types (Akaike weight = 0.99, table S6). For each of those location types, we also found differences in how frequencies and mean durations of visits to locations of those types were affected by distance from home and by a correlation between those quantities. Distance from home had a significant effect only for residential and commercial location types (Akaike weights ≈ 1, table S7), with the frequency and mean duration of visits decreasing and increasing, respectively, with increasing distance from home. Statistical support for a correlation between the frequency and mean duration of visits to locations of each type was only evident for the education and institutions location types (Akaike weights > 0.95, electronic supplementary material, table S6), which had correlations of 0.27 and 0.49, respectively (electronic supplementary material, table S8).

The frequency and mean duration of visits outside the city were best described by a bivariate lognormal distribution with an average frequency of once per 6.4 days, an average mean duration of 4.4 h, and no correlation between those quantities (likelihood ratio test: 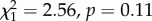) (figure 5).

#### 3.1.3. Neighbourhood comparison

Applying our model fitting and selection procedures separately to interviews from residents of two distinct neighbourhoods, we found strong statistical support for the disaggregated neighbourhood model (*M_M_* + *M_T_*) over the aggregated model (*M_M_*_+_ *_T_*) only for the model subcomponent concerning the effect of distance from home on inclusion in the activity space (ΔAIC*_c_* = 59.24, table S9). Performing a similar comparison of *M_M_* + *M_T_* and *M_M_*_+_ *_T_* by location type (table S10), we found statistical support for differences in the effect of distance from home only for the recreation (ΔAIC*_c_* = 34.86), institutions (ΔAIC*_c_* = 11.66) and others (ΔAIC*_c_* = 13.64) location types (figure 4).

### 3.2. Comparison of model outputs against data

**Figure 6.**
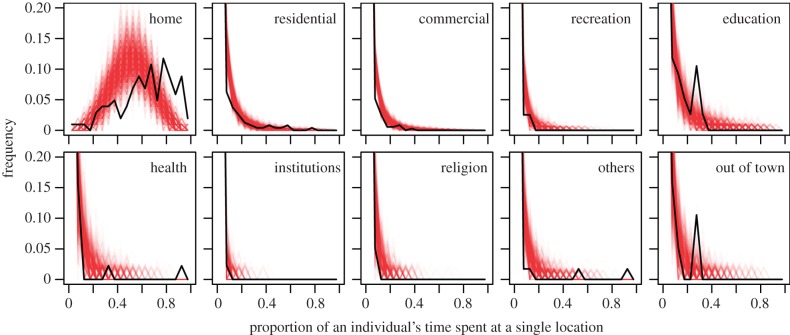
Distributions of the overall proportion of an individual's time spent at a single location of each of several location types. Black lines show the proportion of participant–location pairs in the retrospective interview study for which a given proportion of the participant's time was spent at that location. Red lines show 10^3^ analogous distributions resulting from simulating activity spaces, simulating movement parameters within those activity spaces, and calculating *π* for the same 101 individuals that participated in both parts of the retrospective interview study. (Online version in colour.)

Both empirical and simulated data displayed wide variation in the proportion of time that individuals spent at home (figure 6). However, the fitted model tended to predict that very few people spent a majority of their time at home. For locations other than home, simulated patterns of time allocation were consistent with the empirical pattern that most people spent relatively little time at any single location (i.e. 10% or less). The most significant departures from this pattern in the empirical data were that approximately 10% of study participants who visited an educational location or a location outside of Iquitos spent significantly more time at those locations (approx. 20−30%) than was exhibited in the simulated data. For location types that were relatively infrequently included in one's activity space (i.e. health, institutions, religion, others), the empirical pattern of very few individuals spending a significant amount of their time at a single location of one of those types was consistent with individual replicates of the simulation (figure 6).

**Figure 7.**
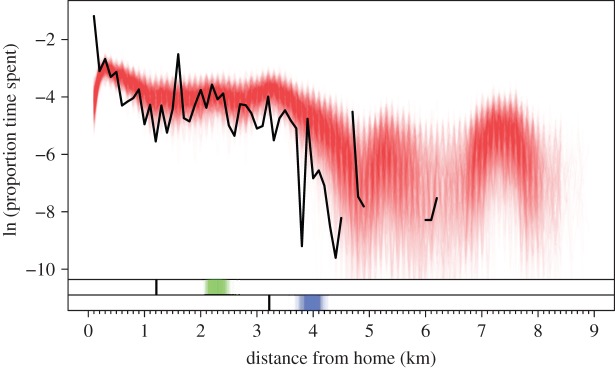
Distribution of time allocation over distance from home, as measured by the retrospective interviews (black) and in 10^3^ simulations of the fitted model (red). The bar directly below the main panel shows the mean distance at which time is allocated for the empirical (black) and each simulated (green) distribution. The bar along the bottom shows the mean distance of locations visited in the empirical (black) and each simulated (blue) activity space. (Online version in colour.)

Simulated data also captured some patterns of time allocation over a range of distances from home but were deficient in other ways. One of the primary deficiencies of the simulated data was that they under-predicted the proportion of time allocated within 100 m of an individual's home (figure 7). That time was instead allocated elsewhere, thereby shifting the simulated patterns of time allocation at distances beyond 100 m above the empirical pattern at those distances. The simulated data captured the mean pattern of time allocation between distances of approximately 100 m to 5 km relatively well otherwise, but they appeared to under-represent the variability in time allocation at different distances (i.e. the simulated patterns were relatively smooth compared with the empirical pattern). The simulations also somewhat over-predicted time allocated beyond 5 km, but in individual replicates this discrepancy was not as severe as it appeared in the ensemble. Examining the mean distance from home of where time was allocated (figure 7, green) and the mean distance of locations visited (figure 7, blue), it appeared that over-predicting the distance from home at which time was allocated resulted from both over-predicting the distance of locations that were visited and over-predicting the frequency and duration of visits to faraway locations.

## 4. Discussion

We developed a new mathematical framework for simulating movement of individual people at fine spatial scales in an urban environment. This framework has advantages over existing alternatives in that it is specified at the scale of individual buildings or lots, it allows for *a priori* simulation of both where individuals go and how they allocate their time, and it can be fitted to a variety of data sources about human movement at fine scales. It is also more general than other simulation models of fine-scale urban movement that are structurally sensitive to details of specific locations. Instead, our framework represents a minimal model for realistically simulating patterns of individual time allocation, yet it is based on conventional mathematical tools that make it readily extensible for various applications where additional detail is warranted.

As a case study for this new framework, we fitted candidate versions of such a model, each with a different level of detail, to a set of 157 interviews of residents of Iquitos, Peru. In doing so, we found that location type and distance from study participants' homes were important factors in determining which locations comprised their activity spaces and how they allocated time across those locations. Simulations of the best-supported model provided a realistic description of aggregate patterns of time allocation, thereby validating the utility of this framework for its intended purpose of realistically simulating activity spaces and time allocation therein for a synthetic population. The attainment of this best-supported model thus represents a significant advance in realistically simulating fine-scale human movement in Iquitos, where empirical and modelling studies of dengue virus epidemiology are ongoing, and a starting point for simulating fine-scale human movement in other resource-poor urban environments. The availability of our code at https://github.com/TAlexPerkins/ActivitySpace_JRSI2014 should facilitate adaptation of the model to other cities and extension of the model around additional complexities that other datasets may be well suited to address.

### 4.1. Interpretations of model and results

#### 4.1.1. Activity space composition

Our framework posits that activity space composition is dynamic and that different classes of locations within the activity space turn over at different rates. Currently, we are unable to examine this process of turnover empirically, because we do not possess data appropriate for doing so. Ideally, data to address this question would consist of records of when individuals started and stopped visiting locations over a span of months or even years. Nonetheless, positing such a process provides us with a set of first principles from which to derive candidate models for the distribution of the total number of locations that one visits on a time scale that is short relative to that of activity space turnover. By comparing three theoretically motivated candidate models, we found support for a negative binomial distribution of this quantity. Even for applications in which our model is not used in its entirety, knowledge of the distribution of the number of locations visited should be useful for informing realistic degree distributions for network models [38,39].

Another aspect of activity space composition that our framework accounts for is that some location types appear in activity spaces in proportions much greater (or smaller) than their representation in the city. Indeed, we found clear support for this hypothesis in Iquitos, with 71% of the locations that study participants frequented belonging to the commercial, residential, or recreational location types. An implication of this result is that models that do not incorporate information about location types may miss important aspects of the structure of human movement at intra-urban scales. Although the importance of location types has been recognized in the time-space geography literature, models of human movement developed for coarser scales (e.g. [40,41]) generally ignore information about location types and may therefore perform poorly when applied at increasingly finer scales where location-type composition is highly heterogeneous. Incorporating information about location types depends not only on the details of a model, however, but also on the availability of comprehensive and reliable spatial data, which in many cases will either not be available or will have some uncertainty about location type designation. Although such information may not be widely available at present for many cities and countries, it is becoming increasingly available where research programmes are ongoing and is likely to become increasingly so in coming years, given advances in satellite image processing [42]. For certain applications in epidemiology, this information will be indispensible, as risk is greater at some location types than others [43–45].

We also found a clear effect of distance from home on the probability that a location was included in one's activity space. As a result, networks of co-location or contact generated by a process such as the one described herein have a distinct spatial dimension, which has a number of implications for network topology and for the spread of infectious diseases on such networks [46,47]. Importantly, for seven of the 10 location types we considered, study participants from two geographically distinct neighbourhoods possessed statistically indistinguishable values of the parameters that govern the effect of distance from home on inclusion of a location in one's activity space. This suggests that differences in the spatial distribution of locations within and around a neighbourhood, rather than possible social or economic differences, drive differences in the realized distributions of distances over which activity space locations of these seven types are found. One implication of this result is that realized distributions of locations visited may not be comparable across different spatial contexts, as those differences could reflect differences in the distribution of locations rather than differences in the underlying movement process. On the other hand, this result suggests that the capacity of a model like ours to extrapolate to other neighbourhoods in Iquitos, and perhaps elsewhere [48], appears promising provided that comprehensive spatial data on location types are available.

#### 4.1.2. Time allocation within the activity space

Location type was also a major factor in determining how much time individuals spent at locations in their activity spaces. Distance from home, on the other hand, only had an impact on the frequency and mean duration of visits to locations in the residential and commercial categories. These results again highlight the value that spatial data on location types can add to simulations of individual activity spaces, although a number of refinements could still be made to improve the model's capacity to simulate activity spaces that are more quantitatively consistent with empirical data. Indeed, accurately quantifying heterogeneity in the time that individuals spend at locations has the potential to affect forecasts of epidemic behaviour and predictions about the conditions under which a disease will persist [49].

### 4.2. Model limitations and extensions

One important extension of the framework will be identifying how differences in individual attributes such as age, sex and occupation can be used to deterministically account for variation in the movement behaviours of different individuals—for which there is evidence in our study population [9]. Our goal here, however, was not to describe every possible detail that could affect human movement, but to develop a mathematically cohesive description of how five distinct aspects of movement combine to generate patterns of time allocation. Rather than predicting the whereabouts of a single individual, the most reasonable use of our fitted model in its present form is the simulation of patterns of time allocation aggregated over many individuals in a synthetic population. Indeed, our model is suited for this purpose because it explicitly accounts for variation in movement behaviours among the diverse individuals in the study population. In applications in which certain attributes are of particular interest (e.g. age in transmission models of immunizing pathogens), model parameters could be specified as functions of those variables and fitted to data.

Although our results clearly show that location type and distance from home are important determinants of activity space composition, the reality is that other factors likely play a role, too. One such factor that we have omitted is the existence of correlations between locations visited by cohabitants or members of certain social groups [50,51], which are likely to be of importance to the transmission of many infectious diseases [52–54]. We also assume no spatial correlation among locations in one's activity space. One factor that probably generates such patterns is that locations other than home, such as the workplace, could serve as anchor points for making choices about other locations to visit [55]. Extending our methodology to account for this phenomenon is possible, but doing so will require empirically discerning (i.e. adapting the SSI) and statistically describing (i.e. identifying associated factors and quantifying variation) which locations serve as anchor points and which are chosen secondarily. One approach to describing which locations act as anchor points could involve gathering data on which location types tend to be visited following visits to other location types, with likely anchor points being location types frequently visited following time at home [56, figs 5.7 and 5.15]. The choice of locations visited secondarily to those anchor points might then be better informed by distance from the anchor than by distance from home. It also may be worthwhile to consider differences in the identities of anchor locations for individuals of different ages, occupations, etc., as well as whether certain types of locations tend to be associated with anchor locations of certain other types.

For applications in which sequences of movements, or ‘trip chains’ [57], are of interest, the movement process we assume could benefit from a number of elaborations. As much of time-space geography is concerned with such sequences rather than with aggregate distributions of time, a wealth of factors have been proposed that could be incorporated into our framework. Examples include elements of the *Q* matrix that are specific in various ways to different origin–destination pairs [58], an increased probability of returning home with increasing trip length [59], and elements of *Q* that depend on day of week [60] or time of day [61]. Given a proposal for a more detailed formulation of *Q*, existing theory of Markov processes could be used to calculate likelihoods of parameter values given data on sequences of movement. Such realistic accounting of trip chains could also allow for consideration of time in motion between locations, which is of specific interest in some applications and of implicit interest in others vis-à-vis the fact that time in motion reduces time spent at locations in the activity space.

## Supplementary Material

Supplementary tables and figures

## Notes

The study protocol for research with human subjects was approved by the University of California at Davis (Protocol 2007–15244) and Naval Medical Research Unit No. 6 (NAMRU-6 Protocol NMRCD.2007.0007) Institutional Review Boards (IRBs) in compliance with all US Federal and Peruvian regulations governing the protection of human subjects. The NAMRU-6 IRB included Peruvian representation and was registered with the Peruvian Regulatory Agency for Clinical Trials with the number RCEI-78. IRB authorization agreements were established between the University of California at Davis and the University of Illinois at Urbana–Champaign, Emory University, and San Diego State University and between the NAMRU-6 and Tulane University. The protocol was reviewed and approved by the Loreto Regional Health Department, which oversees health research in Iquitos. A strict protocol for data storage (in a secure MySQL database) and management was followed.
