# Supplementary material for: Theory and data for simulating fine-scale human movement in an urban environment
Source: J R Soc Interface. 2014 Oct 6;11(99):20140642. doi: 10.1098/rsif.2014.0642 (PMC4233749; doi:10.1098/rsif.2014.0642)
Supplement: Supplementary tables and figures [file rsif20140642supp1.pdf]

Supporting Information for

Theory and data for simulating fine-scale human movement in an urban environment

T. Alex Perkins*†[1,2], Andres J. Garcia[3,4], Valerie A. Paz-Soldán[5], Steven T. Stoddard[2], Robert C. Reiner, Jr.[1,2], Gonzalo Vazquez-Prokopec[6], Donal Bisanzio[6], Amy C. Morrison[2], Eric S. Halsey[7], Tadeusz J. Kochel[7], David L. Smith[1,8,9], Uriel Kitron[1,6], Thomas W. Scott[1,2], Andrew J. Tatem[1,10,11]

[1] Fogarty International Center, National Institutes of Health, Bethesda, MD, USA

[2] Department of Entomology and Nematology, University of California, Davis, CA, USA

[3] Emerging Pathogens Institute, University of Florida, Gainesville, FL, USA

[4] Department of Geography, University of Florida, Gainesville, FL, USA

[5] Department of Global Health Systems and Development, Tulane University School of Public Health and Tropical Medicine, New Orleans, LA, USA

[6] Department of Environmental Sciences, Emory University, Atlanta, GA, USA

[7] United States Naval Medical Research Unit No. 6, Lima, Peru

[8] Department of Epidemiology, Johns Hopkins Bloomberg School of Public Health, Baltimore, MD, USA

[9] Malaria Research Institute, Johns Hopkins Bloomberg School of Public Health, Baltimore, MD, USA

[10] Department of Geography and Environment, University of Southampton, Southampton, UK

[11] Flowminder Foundation, Stockholm, Sweden

*Author for correspondence; taperkins@nd.edu

†Current address: Department of Biological Sciences and Eck Institute for Global Health, University of Notre Dame, Notre Dame, IN, USA

Tables

Table S1: Occupations of study participants. Separate distributions are shown for study participants for whom there are data about time at home versus time at other locations, as well as in aggregate, from participants residing in the Maynas neighborhood and from participants residing in the Tupac neighborhood.

	Time at home			Time away			All		
Occupation	Maynas	Tupac	Total	Maynas	Tupac	Total	Maynas	Tupac	Total
Student	27	13	40	34	17	51	38	20	58
Housewife	15	16	31	16	24	40	18	25	43
Salesperson	7	5	12	6	4	10	8	5	13
Tradesperson	7	3	10	6	4	10	7	4	11
Transportation	4	1	5	4	1	5	5	2	7
Health/Education	2	2	4	2	3	5	2	3	5
Skilled worker	0	4	4	0	4	4	0	5	5
Unemployed	4	1	5	3	1	4	4	1	5
Unskilled worker	2	2	4	2	2	4	2	2	4
Military/Police	2	0	2	1	0	1	2	0	2
Retired	2	0	2	1	0	1	2	0	2
Office worker	2	0	2	2	0	2	2	0	2

Table S2: Details of the model selection procedure specific to each aspect of movement.

Aspect of movement	Model selection procedure
Number of locations	We compared the three candidate distributions using Akaike weights.
Location types	We compared multinomial distributions in which different levels of detail about location type were considered, using Akaike weights to compare models chosen via the backward elimination routine described in the main text.
Distance of locations from home	We first used a likelihood ratio test to determine whether each $\eta_\mathcal{T} \neq 1$ in eqn. (3). We then chose models with different levels of detail about location-type groupings via backward elimination and compared them with Akaike weights.
Frequency and mean duration of visits to one's home and to locations outside the city	We used a likelihood ratio test to determine whether each $\rho \neq 0$ was supported.
Frequency and mean duration of visits to locations in the city	We fit eight models with all combinations of correlation between frequency and mean duration and with separate dependencies on distance from home on each. After selecting a best-fit model from those eight based on AIC_c minimization, we compared best-fit models with Akaike weights across levels of detail about location type chosen by backward elimination.

Table S3: Comparison by AIC_c of different distributions of activity space size ($n = 138$). Cells with Akaike weights are darkened proportionally.

Distribution	k	AIC_c	$dAIC_c$	Akaike weight
Negative binomial	2	843.4	0	1
Poisson	1	922.7	79.23	6.236e-18
Geometric	1	975.7	132.3	1.878e-29

Table S4: Comparison by AIC_c of models in which there are different numbers of location-type groups with distinct probabilities that a location in one's activity space is of a given location type ($n = 138$). Cells with Akaike weights are darkened proportionally.

Number of location-type groups	k	AIC_c	$dAIC_c$	Akaike weight
6	6	2296	0	0.6997
7	7	2299	2.496	0.2009
8	8	2301	4.778	0.06416
9	9	2304	7.18	0.01931
5	5	2304	7.56	0.01597
4	4	2318	21.19	1.751e-05
3	3	2418	121.4	3.045e-27
2	2	2437	140.4	2.322e-31
1	1	3339	1043	2.727e-227

Table S5: Comparison by AIC_c of models in which there are different numbers of location-type groups with distinct relationships between distance from one's home and the probability that a location at that distance is included in one's activity space ($n = 1402$). Cells with Akaike weights are darkened proportionally.

Number of location-type groups	k	AIC_c	$dAIC_c$	Akaike weight
8	14	1.431e+04	0	1
5	9	1.436e+04	49.48	1.798e-11
6	10	1.436e+04	51.08	8.091e-12
7	12	1.437e+04	53.98	1.898e-12
4	7	1.439e+04	74.94	5.333e-17
3	6	1.44e+04	86.24	1.876e-19
2	4	1.444e+04	126.8	2.955e-28
1	2	1.475e+04	442.4	8.572e-97

Table S6: Comparison by AIC_c of models in which there are different numbers of location-type groups with distinct relationships between distance from one's home and the average frequency and duration of visits to a location ($n = 1654$). Cells with Akaike weights are darkened proportionally.

Number of location-type groups	k	AIC_c	$dAIC_c$	Akaike weight
8	44	7866	0	0.9905
7	39	7875	9.284	0.009548
6	34	7904	38.67	3.962e-09
5	30	7927	61.09	5.388e-14
4	26	8011	145.5	2.498e-32
3	22	8028	161.8	7.292e-36
2	13	8037	171.3	6.424e-38
1	8	8095	229.2	1.683e-50

Table S7: For each location type, Akaike weights of candidate models for separate effects of distance from home (δ) on frequency ($\mu_f(\delta)$ or μ_f) and mean duration ($\mu_d(\delta)$ or μ_d) of visits and a correlation between frequency and mean duration of visits ($\rho \neq 0$ or $\rho = 0$). Akaike weights are comparable across columns within a given row, and the entries in each row sum to unity. Each cell is darkened proportional to the Akaike weight of the model it represents.

	$\rho \neq 0$				$\rho = 0$			
	$\mu_f(\delta)$		μ_f		$\mu_f(\delta)$		μ_f	
Location type	$\mu_d(\delta)$	μ_d	$\mu_d(\delta)$	μ_d	$\mu_d(\delta)$	μ_d	$\mu_d(\delta)$	μ_d
Residential	0.57	0.002	2e-08	3e-12	0.43	0.0002	3e-09	4e-12
Commercial	0.004	3e-07	5e-08	2e-13	1	2e-07	2e-08	6e-15
Recreation	0.02	0.04	0.15	0.36	0.01	0.03	0.12	0.27
Education	0.01	0.08	0.14	0.72	0.0006	0.004	0.006	0.03
Health	0.001	0.02	0.02	0.24	0.004	0.06	0.06	0.6
Institutions	0.006	0.07	0.09	0.83	4e-06	5e-05	5e-05	0.0005
Religion	0.008	0.03	0.11	0.21	0.02	0.05	0.17	0.41
Others	0.002	0.02	0.02	0.2	0.007	0.08	0.06	0.61

Table S8: For each location type, maximum-likelihood value of the correlation coefficient ρ of frequency and mean duration of visits on a log scale, under candidate models with different effects of distance from home (δ) on frequency ($\mu_f(\delta)$ or μ_f) and mean duration ($\mu_d(\delta)$ or μ_d) of visits. Each cell is darkened proportional to the Akaike weight of the model it represents, in the same way as in Table S7.

	$\mu_f(\delta)$		μ_f	
Location type	$\mu_d(\delta)$	μ_d	$\mu_d(\delta)$	μ_d
Residential	0.13	0.15	0.13	0.067
Commercial	-0.066	-0.067	-0.086	-0.13
Recreation	-0.12	-0.12	-0.12	-0.12
Education	0.26	0.27	0.27	0.27
Health	0.1	0.1	0.1	0.1
Institutions	0.5	0.5	0.5	0.49
Religion	0.17	0.18	0.18	0.15
Others	0.015	0.015	0.016	0.015

Table S9: Comparison of model subcomponents fit without consideration of whether interviewees reside in the Maynas or Tupac neighborhood (M_{M+T}) against model subcomponents fit separately to interviews from residents of those neighborhoods (M_M and M_T). Each neighborhood-specific model M_M and M_T represents the best-fit model as determined by the model selection procedures for M_{M+T} described in the main text. We assessed support for differences between neighborhoods with respect to each model subcomponent by the difference between the AIC_c of $M_M + M_T$ and that of M_{M+T} (which we refer to as ΔAIC_c in the table). Consistent with guidelines for comparison of a pair of non-nested models recommended on page 123 of Burnham and Anderson (1998), we consider there to be strong support for one model or other other if $|\Delta AIC_c| > 10$.

	$M_{\mathrm{M+T}}$		$M_{\mathrm{M}} + M_{\mathrm{T}}$			
Model subcomponent	LL	k	LL	k	ΔAIC_c	Best model
Number of locations	-419.7	2	-420.6	3	-4.184	–
Locations of a given type	-1142	6	-1141	10	-10.84	M_{M+T}
Locations of a given distance	-7142	14	-7098	27	59.24	$M_M + M_T$
Frequency and duration of visits (home)	-105.7	5	-103.5	10	4.398	–
Frequency and duration of visits (away)	-3888	44	-3846	85	-18.85	M_{M+T}

Table S10: Comparison of the model subcomponent for choosing locations as a function of distance from home fit without consideration of whether interviewees reside in the Maynas or Tupac neighborhood (M_{M+T}) against model subcomponents fit separately to interviews from residents of those neighborhoods (M_M and M_T). Each neighborhood-specific model M_M and M_T represents the best-fit model as determined by the model selection procedures for M_{M+T} described in the main text. We assessed support for differences between neighborhoods with respect to each model subcomponent by the difference between the AIC_c of $M_M + M_T$ and that of M_{M+T} (which we refer to as ΔAIC_c in the table). Consistent with guidelines for comparison of a pair of non-nested models recommended on page 123 of Burnham and Anderson (1998), we consider there to be strong support for one model or other other if $|\Delta AIC_c| > 10$.

	$M_{\text{M+T}}$		$M_{\text{M}} + M_{\text{T}}$			
Location type	LL	k	LL	k	ΔAIC_c	Best model
Residential	-1952	2	-1949	4	2.019	–
Commercial	-2829	2	-2829	4	-2.952	–
Recreation	-761.6	2	-743.2	3	34.86	$M_M + M_T$
Education	-441.8	2	-439.9	4	-0.1652	–
Health	-196.2	1	-192.3	3	3.984	–
Institutions	-240.4	2	-232.6	4	11.66	$M_M + M_T$
Religion	-216	1	-215.8	2	-1.635	–
Others	-504.3	2	-496.5	3	13.64	$M_M + M_T$

Figures

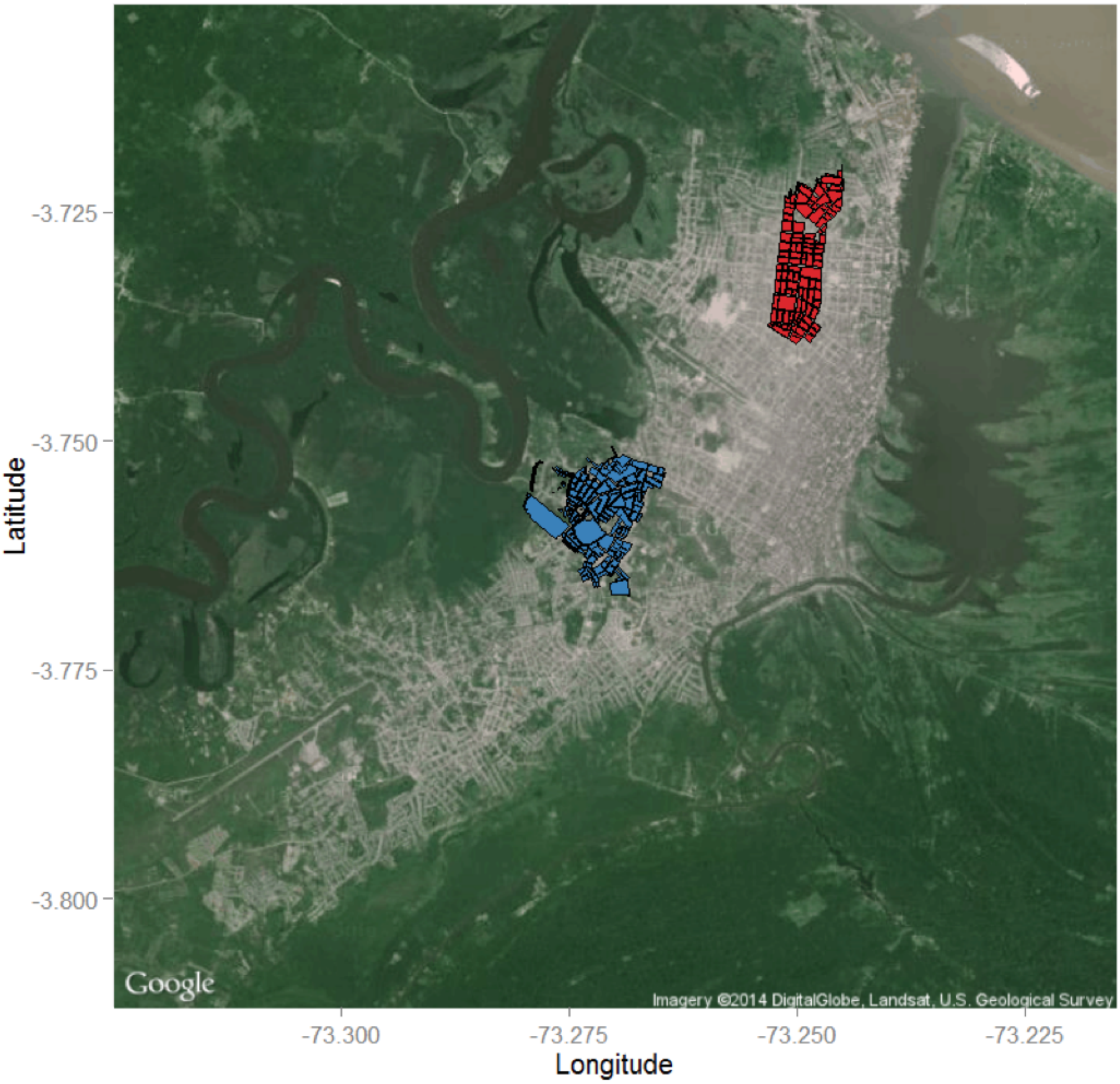

Figure S1: Map of Iquitos showing the two neighborhoods in which study participants reside. The Maynas neighborhood is shown in blue and Tupac is shown in red.

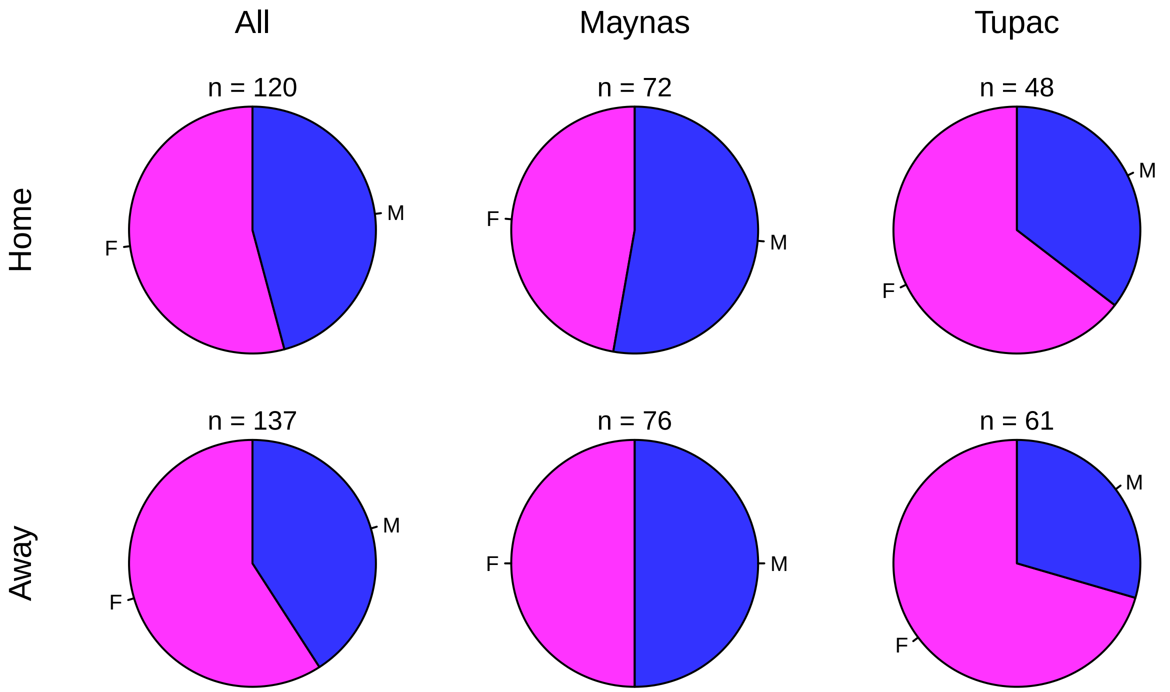

Figure S2: Sex distributions of study participants. Separate distributions are shown for study participants for whom there are data about time at home versus time at other locations, as well as in aggregate, from participants residing in the Maynas neighborhood, and from participants residing in the Tupac neighborhood.

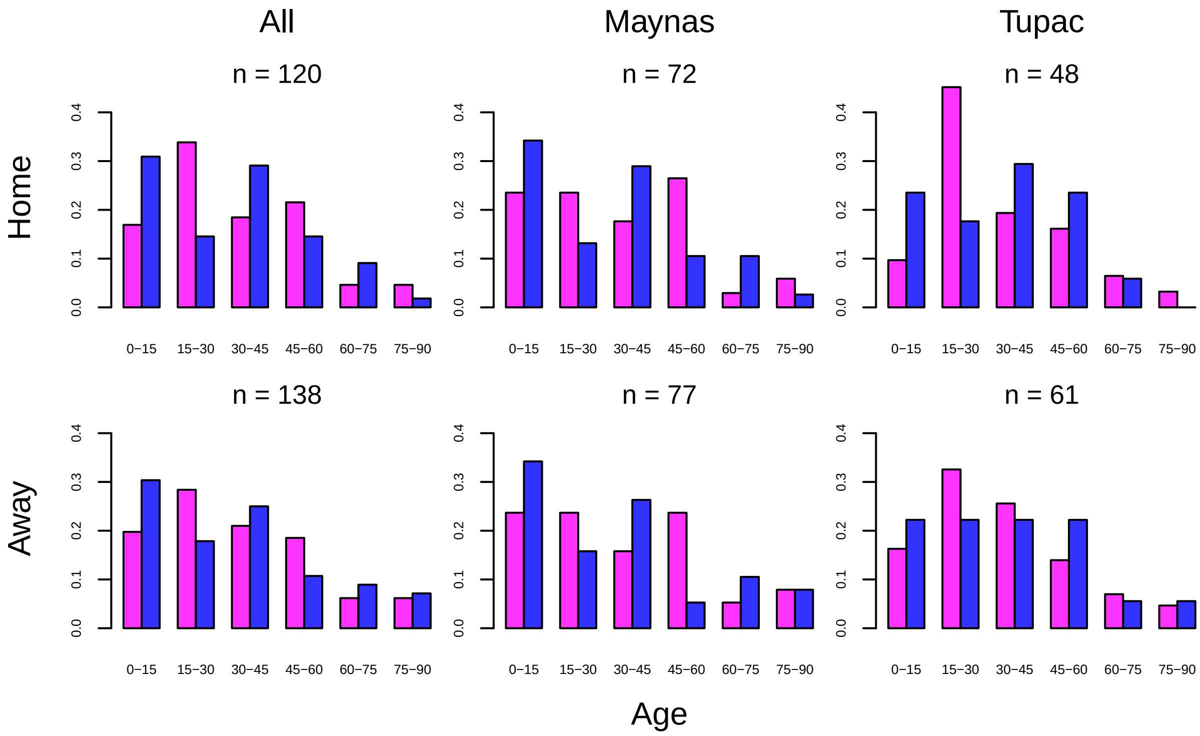

Figure S3: Age distributions of study participants by sex. Separate distributions are shown for study participants for whom there are data about time at home versus time at other locations, as well as in aggregate, from participants residing in the Maynas neighborhood, and from participants residing in the Tupac neighborhood.

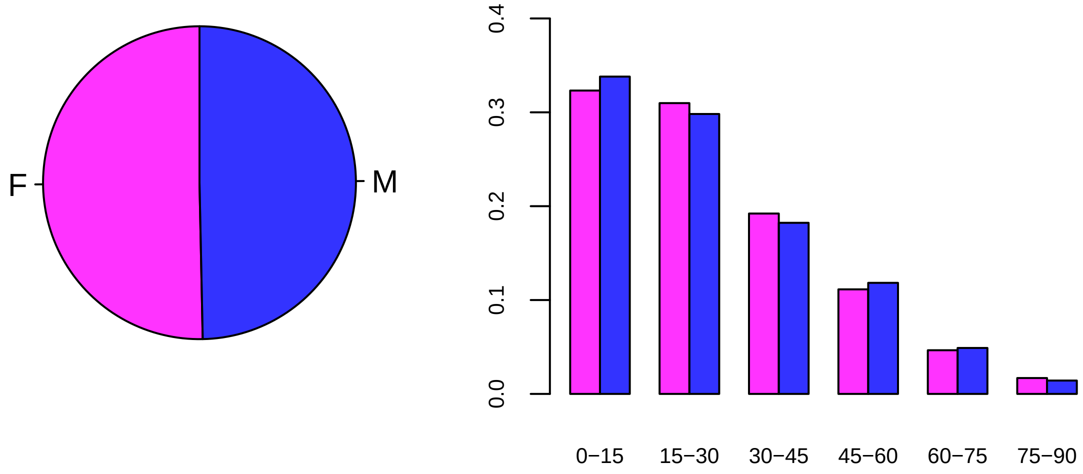

Figure S4: Sex and age-by-sex distributions from government census data for Iquitos.

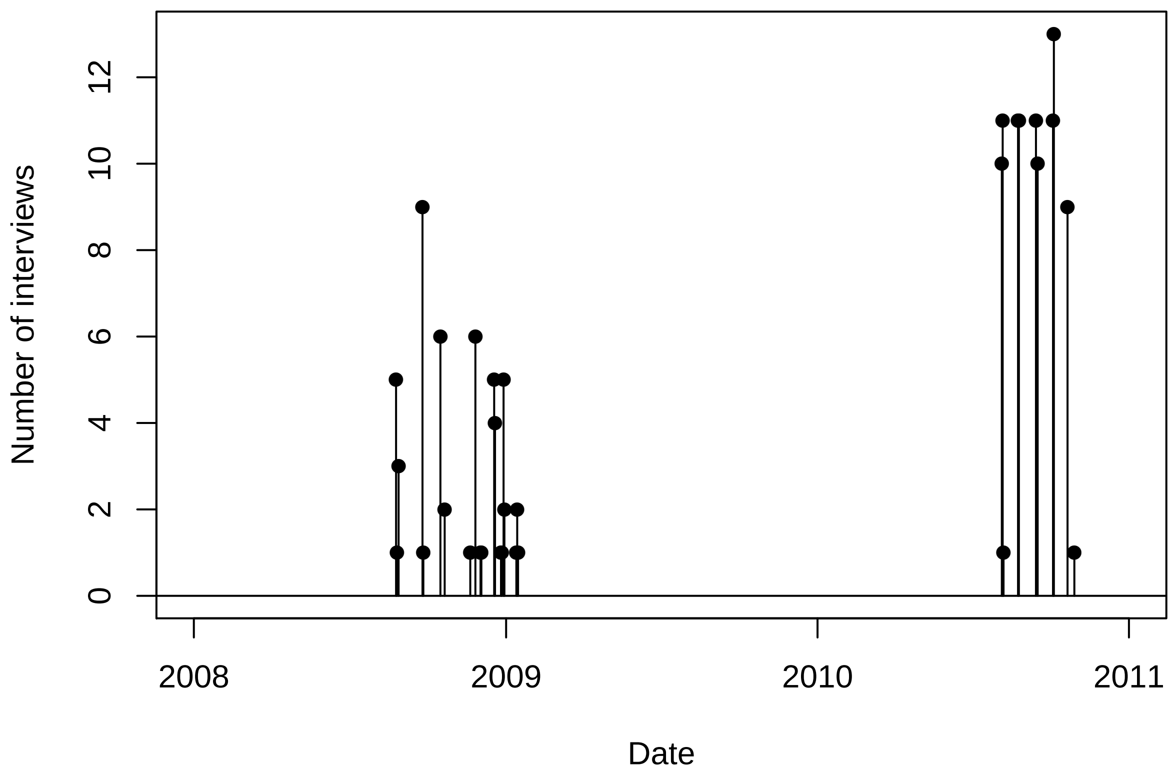

Figure S5: Number of retrospective interviews performed by date. Each number on the horizontal axis refers to January 1 of the indicated year.
